# 1-Ethyl-4′-(1*H*-indol-3-ylcarbon­yl)-1′-methyl-2,2′′-dioxodi­spiro­[indoline-3,2′-pyrrolidine-3′,3′′-indoline]-4′-carbo­nitrile dimethyl sulfoxide monosolvate

**DOI:** 10.1107/S1600536813020485

**Published:** 2013-07-27

**Authors:** S. Antony Inglebert, Yuvaraj Arun, K. Sethusankar, Paramasivam T. Perumal

**Affiliations:** a Sri Ram Engineering College, Chennai 602 024, India; b Organic Chemistry Division, Central Leather Research Institute, Adyar, Chennai 600 020, India; c Department of Physics, RKM Vivekananda College (Autonomous), Chennai 600 004, India

## Abstract

In the title compound, C_31_H_25_N_5_O_3_·C_2_H_6_OS, the three indole/indoline units are all essentially planar with maximum deviations of 0.0172 (3), 0.053 (2) and 0.07 (2) Å. The pyrrolidine ring adopts an envelope conformation with the C atoms bearing the 1-ethyl-2-oxo­indole substituent (in which the five-membered ring adopts a twisted conformation) as the flap. The dimethyl sulfoxide solvent mol­ecule is disordered over two positions, with an occupancy factor ratio of 0.871 (4):0.129 (4). The solvent components are linked to the heterocyclic mol­ecule *via* C—H⋯O and C—H⋯S hydrogen bonds. In the crystal, the solvent components are linked to the heterocyclic molecule *via* C—H⋯O and C—H⋯S inter­actions, forming *R*_2_^2^(10) ring motifs. The mol­ecules are further connected into a chain along the *a-*axis direction *via* N—H⋯O hydrogen bonds.

## Related literature

For applications of indole derivatives, see: Barden (2011 ▶). For puckering parameters, see: Cremer & Pople (1975 ▶). For bond-length data, see: Allen *et al.* (1987 ▶). For graph-set notation, see: Bernstein *et al.* (1995 ▶).
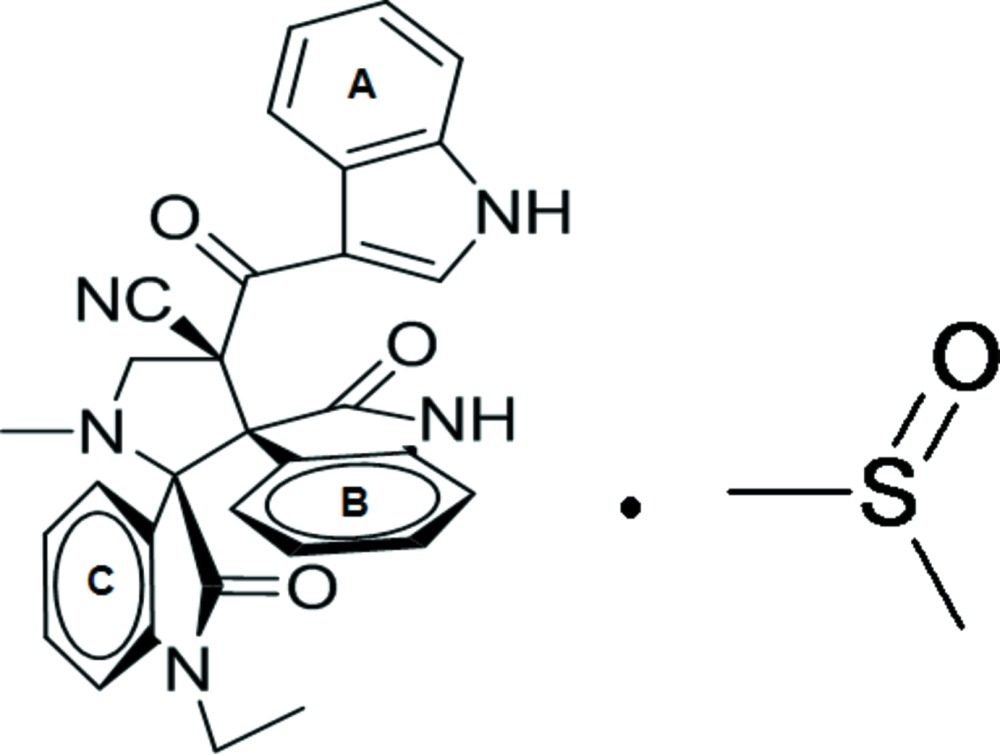


## Experimental

### 

#### Crystal data

C_31_H_25_N_5_O_3_·C_2_H_6_OS
*M* *_r_* = 593.70
Orthorhombic, 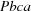

*a* = 14.078 (5) Å
*b* = 20.416 (5) Å
*c* = 20.789 (5) Å
*V* = 5975 (3) Å^3^
*Z* = 8
Mo *K*α radiation
μ = 0.16 mm^−1^
*T* = 295 K
0.30 × 0.20 × 0.20 mm

#### Data collection

Bruker Kappa APEXII CCD diffractometer
Absorption correction: multi-scan (*SADABS*; Bruker, 2008 ▶) *T*_min_ = 0.955, *T*_max_ = 0.970
26724 measured reflections
5139 independent reflections
3447 reflections with *I* > 2σ(*I*)
*R*_int_ = 0.035

#### Refinement

*R*[*F* ^2^ > 2σ(*F* ^2^)] = 0.042
*wR*(*F* ^2^) = 0.120
*S* = 1.03
5139 reflections
431 parameters
48 restraints
H atoms treated by a mixture of independent and constrained refinement
Δρ_max_ = 0.25 e Å^−3^
Δρ_min_ = −0.27 e Å^−3^

### 

Data collection: *APEX2* (Bruker, 2008 ▶); cell refinement: *SAINT* (Bruker, 2008 ▶); data reduction: *SAINT*; program(s) used to solve structure: *SHELXS97* (Sheldrick, 2008 ▶); program(s) used to refine structure: *SHELXL97* (Sheldrick, 2008 ▶); molecular graphics: *ORTEP-3 for Windows* (Farrugia, 2012 ▶) and *Mercury* (Macrae *et al.*, 2008 ▶); software used to prepare material for publication: *SHELXL97* and *PLATON* (Spek, 2009 ▶).

## Supplementary Material

Crystal structure: contains datablock(s) global, I. DOI: 10.1107/S1600536813020485/rk2408sup1.cif

Structure factors: contains datablock(s) I. DOI: 10.1107/S1600536813020485/rk2408Isup2.hkl

Additional supplementary materials:  crystallographic information; 3D view; checkCIF report

## Figures and Tables

**Table 1:** Hydrogen-bond geometry (Å, °)

*D*—H⋯*A*	*D*—H	H⋯*A*	*D*⋯*A*	*D*—H⋯*A*
N4—H4*A*⋯O4	0.84 (3)	1.89 (3)	2.714 (3)	166 (2)
N1—H1*A*⋯O2^i^	0.84 (3)	2.10 (3)	2.887 (3)	155 (2)
C22—H22⋯S1^ii^	0.93	2.85	3.717 (3)	157
C32—H32*A*⋯O3^iii^	0.96	2.60	3.219 (4)	123

Symmetry codes: (i) 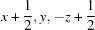; (ii) 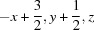; (iii) 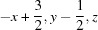.

**Fig. 1.**
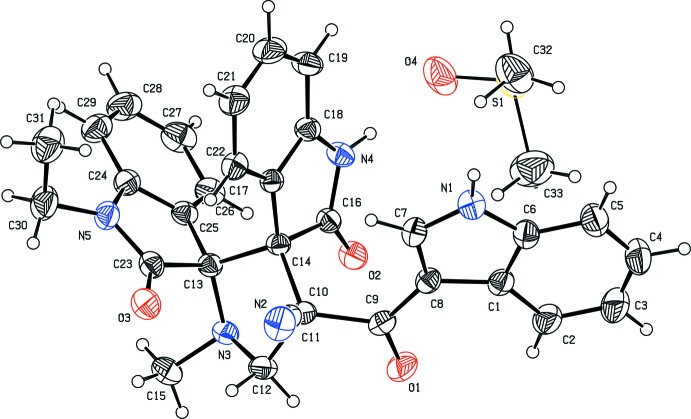
The part of molecular structure of the title compound, showing the atom numbering scheme. Displacement ellipsoids are drawn at 30% probability level. H atoms are present as a small spheres of arbitary radius. Only major moiety of solvent is shown.

**Fig. 2.**
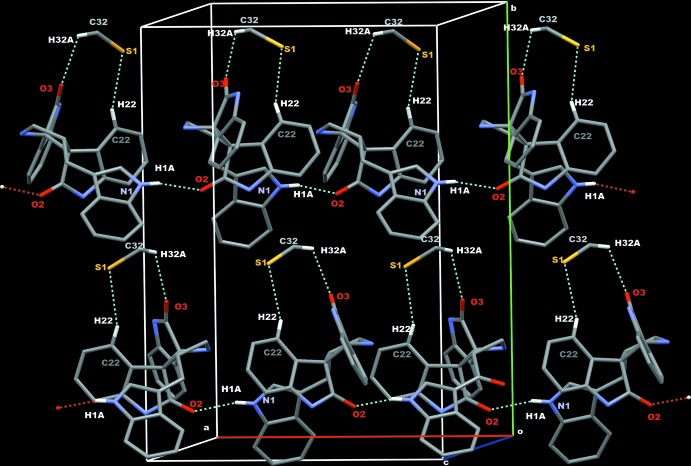
The packing structure of the title compound viewed along the c axis. H atoms not included in H-bonding have omited for clarity.

## supplementary crystallographic information

### Comment

Indole containing compounds are best known for their medicinal properties in the pharmaceutical industry. In modern times, analogs based on indole are significant players in a diverse array of markets such as dyes, plastics, agriculture, vitamin supplements, over-the-counterdrugs, flavour enhancers and perfumery (Barden, 2011).

In the crystal structure of title compound, C_31_H_25_N_5_O_3_^.^C_2_H_6_OS, (Fig. 1), there is a dispiro centers system, which consists of two oxindole rings, an indole ring and a pyrrolidine ring. In crystals organic heterocycles moiety and solvent molecules connected by two intermolecular C–H···S and C–H···O hydrogen bonds (Table 1). The dimethyl sulfoxide solvent molecule is disordered over two positions with site occupancy factors 0.871 (4) and 0.129 (4).

The pyrrolidine ring (N3/C12/C13/C14/C10) adopts an envelope conformation with puckering parameters, q_2_ = 0.402 (2)Å, φ(2) = 331.4 (3)°, and with atom C13 deviating by 0.256 (2)Å from the mean plane passing through the rest of the ring atoms (Cremer & Pople, 1975). The carbonitrile group is nearly perpendicular to pyrrolidine ring, as indicated by the torsion angle C12-C10-C11-N2 = 87.4 (2)°. The indole system *A* makes dihedral angles of 53.13 (15)° and 86.11 (6)° with the oxindole ring systems *B* & *C*, respectively. It clearly shows that ring systems *A* and *C* are almost perpendicular to each other. Also the dihedral angle between oxindole *B* and *C* is 54.40 (5)°. The twisted conformation of the five membered pyrrole ring in oxindole ring system *C* is observed through the puckering analysis [q_2_ = 0.074 (2) Å and φ(2) = 277.1 (18)°].

The indole and oxindole ring systems *A*, *B* and *C* are planar with maximum deviations of 0.0172 (3)Å, 0.053 (2)Å and 0.07 (2)Å for the atoms C4, C14 and C26 from the LS planes. The bond lengths of O2-C16, C16-N4 in oxindole unit *B* and O3-C23, C23-N5 in oxindole unit *C* show electron delocalization over atoms O2, C16, N4, O3, C23 and N5.

The cyano bond distance C11≡N2 agrees well with the reported value of 1.138 (7)Å (Allen *et al.*, 1987). The sum of the angles around atom N5 (359.86°) is in accordance with *sp^2^* hybridization, whereas the sum of the angles around atom N3 (337.75°) is in accordance with *sp^3^* one. The oxygen atoms attached to C16 and C23 are coplanar with the oxindole ring system *B* & *C* as indicated by the torsion angles O2-C16-N4-C18 = -177.3 (2)° and O3-C23-N5-C24 = -176.3 (2)°, respectively.

In the crystal structure, pairs of molecules are linked by intermolecular C–H···O and C–H···S hydrogen bonds to generate *R*^2^_2_(10) ring motifs (Bernstein *et al.*, 1995). The molecules are further connected into a chain along the *a* axis *via* N–H···O intermolecular hydrogen bonds. The packing view of the title compound is shown in Fig. 2. (Macrae *et al.*, 2008).

### Experimental

A mixture of 1-ethyl-isatin (1 mmol), sarcosine (1 mmol) and 3-(1*H*-indol-3-yl)-3-oxo-2-(2-oxoindoline-3-ylidene)propane nitrile (1 mmol) were refluxed in ethanol (10 ml). After completion of the reaction as evidenced by *TLC* analysis, the reaction mixture was poured into ice-water, the resulting solid was filtered off and purified by column chromatography using ethyl acetate : petroleum ether (6 : 4) as an eluent to afford pure spirooxiindoles in 82% yield.

### Refinement

Positions of hydrogen atoms were localized from the difference electron density maps and their distances were geometrically constrained. The H atoms bound to the C atoms were treated as riding atoms,with C–H = 0.93Å and *U*_iso_(H) = 1.2*U*_eq_(C) for aromatic, C–H = 0.97Å and *U*_iso_(H) = 1.2*U*_eq_(C) for methylene and C–H = 0.96Å and *U*_iso_(H) = 1.5*U*_eq_(C) for methyl groups. The rotation angles for methyl groups were optimized by least squares. The N bonded H atoms were refined freely.

### Crystal data

**Table:**

C_31_H_25_N_5_O_3_·C_2_H_6_OS	*F*(000) = 2496
*M**_r_* = 593.70	*D*_x_ = 1.320 Mg m^−^^3^
Orthorhombic, *P**b**c**a*	Mo *K*α radiation, λ = 0.71073 Å
Hall symbol: -P 2ac 2ab	Cell parameters from 5139 reflections
*a* = 14.078 (5) Å	θ = 2.2–25.0°
*b* = 20.416 (5) Å	µ = 0.16 mm^−^^1^
*c* = 20.789 (5) Å	*T* = 295 K
*V* = 5975 (3) Å^3^	Block, colourless
*Z* = 8	0.30 × 0.20 × 0.20 mm

### Data collection

**Table:**

Bruker Kappa APEXII CCD diffractometer	5139 independent reflections
Radiation source: fine-focus sealed tube	3447 reflections with *I* > 2σ(*I*)
Graphite monochromator	*R*_int_ = 0.035
ω and φ scans	θ_max_ = 25.0°, θ_min_ = 2.2°
Absorption correction: multi-scan (*SADABS*; Bruker, 2008)	*h* = −16→15
*T*_min_ = 0.955, *T*_max_ = 0.970	*k* = −16→24
26724 measured reflections	*l* = −24→24

### Refinement

**Table:**

Refinement on *F*^2^	Secondary atom site location: difference Fourier map
Least-squares matrix: full	Hydrogen site location: inferred from neighbouring sites
*R*[*F*^2^ > 2σ(*F*^2^)] = 0.042	H atoms treated by a mixture of independent and constrained refinement
*wR*(*F*^2^) = 0.120	*w* = 1/[σ^2^(*F*_o_^2^) + (0.0509*P*)^2^ + 2.1171*P*] where *P* = (*F*_o_^2^ + 2*F*_c_^2^)/3
*S* = 1.03	(Δ/σ)_max_ < 0.001
5139 reflections	Δρ_max_ = 0.25 e Å^−^^3^
431 parameters	Δρ_min_ = −0.27 e Å^−^^3^
48 restraints	Extinction correction: *SHELXL97* (Sheldrick, 2008), Fc^*^=kFc[1+0.001xFc^2^λ^3^/sin(2θ)]^-1/4^
Primary atom site location: structure-invariant direct methods	Extinction coefficient: 0.0015 (2)

### Special details

**Table:**

Geometry. All s.u.'s (except the s.u. in the dihedral angle between two l.s. planes) are estimated using the full covariance matrix. The cell s.u.'s are taken into account individually in the estimation of s.u.'s in distances, angles and torsion angles; correlations between s.u.'s in cell parameters are only used when they are defined by crystal symmetry. An approximate (isotropic) treatment of cell s.u.'s is used for estimating s.u.'s involving l.s. planes.
Refinement. Refinement of *F*^2^ against ALL reflections. The weighted *R*-factor *wR* and goodness of fit *S* are based on *F*^2^, conventional *R*-factors *R* are based on *F*, with *F* set to zero for negative *F*^2^. The threshold expression of *F*^2^ > σ(*F*^2^) is used only for calculating *R*-factors(gt) *etc*. and is not relevant to the choice of reflections for refinement. *R*-factors based on *F*^2^ are statistically about twice as large as those based on *F*, and *R*-factors based on ALL data will be even larger.

### Fractional atomic coordinates and isotropic or equivalent isotropic displacement parameters (Å^2^)

**Table:**

	*x*	*y*	*z*	*U*_iso_*/*U*_eq_	Occ. (<1)
C1	0.65192 (17)	0.06281 (11)	0.33748 (10)	0.0472 (6)	
C2	0.5854 (2)	0.02329 (12)	0.36920 (13)	0.0661 (7)	
H2	0.5206	0.0308	0.3648	0.079*	
C3	0.6188 (3)	−0.02699 (14)	0.40713 (16)	0.0868 (10)	
H3	0.5754	−0.0536	0.4285	0.104*	
C4	0.7148 (3)	−0.03925 (14)	0.41453 (15)	0.0853 (10)	
H4	0.7341	−0.0740	0.4403	0.102*	
C5	0.7820 (2)	−0.00148 (13)	0.38480 (13)	0.0683 (8)	
H5	0.8466	−0.0095	0.3898	0.082*	
C6	0.74840 (18)	0.04948 (11)	0.34678 (10)	0.0501 (6)	
C7	0.73904 (17)	0.13393 (11)	0.27982 (10)	0.0482 (6)	
H7	0.7573	0.1679	0.2527	0.058*	
C8	0.64624 (16)	0.11757 (10)	0.29381 (10)	0.0434 (5)	
C9	0.55927 (17)	0.14783 (11)	0.27187 (10)	0.0470 (6)	
C10	0.56313 (15)	0.20484 (10)	0.22079 (10)	0.0407 (5)	
C11	0.62745 (17)	0.25514 (11)	0.24702 (11)	0.0468 (6)	
C12	0.46423 (16)	0.23568 (11)	0.20902 (11)	0.0503 (6)	
H12A	0.4175	0.2166	0.2378	0.060*	
H12B	0.4663	0.2826	0.2163	0.060*	
C13	0.52858 (15)	0.21989 (10)	0.10596 (10)	0.0399 (5)	
C14	0.59516 (14)	0.18003 (9)	0.15212 (10)	0.0374 (5)	
C15	0.36806 (17)	0.26636 (12)	0.11622 (13)	0.0619 (7)	
H15A	0.3895	0.3107	0.1210	0.093*	
H15B	0.3096	0.2606	0.1393	0.093*	
H15C	0.3580	0.2571	0.0715	0.093*	
C16	0.57207 (16)	0.10561 (10)	0.14492 (10)	0.0413 (5)	
C17	0.69758 (15)	0.18221 (10)	0.13168 (9)	0.0382 (5)	
C18	0.72407 (15)	0.12012 (11)	0.11078 (10)	0.0433 (5)	
C19	0.81171 (18)	0.10731 (13)	0.08455 (12)	0.0598 (7)	
H19	0.8281	0.0655	0.0707	0.072*	
C20	0.87455 (19)	0.15900 (15)	0.07958 (13)	0.0695 (8)	
H20	0.9344	0.1519	0.0619	0.083*	
C21	0.85034 (17)	0.22058 (14)	0.10016 (13)	0.0617 (7)	
H21	0.8940	0.2545	0.0964	0.074*	
C22	0.76217 (16)	0.23289 (12)	0.12629 (10)	0.0482 (6)	
H22	0.7463	0.2748	0.1401	0.058*	
C23	0.57066 (16)	0.28945 (11)	0.09338 (11)	0.0461 (5)	
C24	0.57243 (16)	0.23597 (11)	−0.00259 (11)	0.0480 (6)	
C25	0.52630 (15)	0.19253 (11)	0.03842 (10)	0.0426 (5)	
C26	0.49175 (17)	0.13434 (12)	0.01454 (12)	0.0536 (6)	
H26	0.4580	0.1057	0.0408	0.064*	
C27	0.5084 (2)	0.11921 (15)	−0.05004 (13)	0.0680 (8)	
H27	0.4859	0.0801	−0.0671	0.082*	
C28	0.5577 (2)	0.16173 (16)	−0.08827 (13)	0.0713 (8)	
H28	0.5699	0.1502	−0.1308	0.086*	
C29	0.58995 (18)	0.22098 (14)	−0.06587 (12)	0.0632 (7)	
H29	0.6224	0.2499	−0.0926	0.076*	
C30	0.6331 (2)	0.35405 (14)	0.00144 (14)	0.0722 (8)	
H30A	0.6172	0.3909	0.0288	0.087*	
H30B	0.6025	0.3609	−0.0398	0.087*	
C31	0.7364 (2)	0.35289 (15)	−0.00822 (14)	0.0824 (9)	
H31A	0.7673	0.3436	0.0319	0.124*	
H31B	0.7571	0.3947	−0.0239	0.124*	
H31C	0.7523	0.3196	−0.0390	0.124*	
C32	0.8172 (3)	−0.05035 (18)	0.2089 (2)	0.0967 (14)	0.871 (4)
H32A	0.8741	−0.0559	0.1840	0.145*	0.871 (4)
H32B	0.8265	−0.0684	0.2511	0.145*	0.871 (4)
H32C	0.8028	−0.0045	0.2125	0.145*	0.871 (4)
C33	0.6338 (4)	−0.0748 (3)	0.2305 (3)	0.1150 (16)	0.871 (4)
H33A	0.6239	−0.0284	0.2338	0.173*	0.871 (4)
H33B	0.6547	−0.0916	0.2712	0.173*	0.871 (4)
H33C	0.5753	−0.0957	0.2185	0.173*	0.871 (4)
S1	0.72223 (14)	−0.09108 (5)	0.17075 (6)	0.0784 (5)	0.871 (4)
C32'	0.735 (3)	−0.0416 (12)	0.2131 (13)	0.098 (3)	0.129 (4)
H32D	0.7569	−0.0172	0.1765	0.147*	0.129 (4)
H32E	0.7884	−0.0607	0.2349	0.147*	0.129 (4)
H32F	0.7021	−0.0127	0.2420	0.147*	0.129 (4)
C33'	0.569 (3)	−0.0689 (17)	0.2306 (18)	0.109 (4)	0.129 (4)
H33D	0.5729	−0.0220	0.2331	0.164*	0.129 (4)
H33E	0.5681	−0.0870	0.2732	0.164*	0.129 (4)
H33F	0.5117	−0.0810	0.2084	0.164*	0.129 (4)
S1'	0.6645 (11)	−0.0983 (4)	0.1898 (6)	0.101 (2)	0.129 (4)
N1	0.79922 (17)	0.09409 (9)	0.31071 (10)	0.0512 (5)	
N2	0.67536 (17)	0.29310 (10)	0.27061 (10)	0.0656 (6)	
N3	0.43995 (12)	0.22165 (8)	0.14198 (9)	0.0441 (5)	
N4	0.64895 (14)	0.07659 (10)	0.11969 (9)	0.0475 (5)	
N5	0.59495 (13)	0.29407 (9)	0.03049 (9)	0.0506 (5)	
O1	0.48161 (12)	0.13018 (9)	0.29122 (9)	0.0708 (5)	
O2	0.49819 (11)	0.07829 (7)	0.15947 (8)	0.0540 (4)	
O3	0.57737 (12)	0.33293 (7)	0.13313 (8)	0.0601 (5)	
O4	0.69303 (17)	−0.05270 (9)	0.11415 (10)	0.0955 (7)	
H1A	0.8589 (18)	0.0966 (11)	0.3091 (12)	0.054 (8)*	
H4A	0.6529 (17)	0.0356 (13)	0.1161 (12)	0.062 (8)*	

### Atomic displacement parameters (Å^2^)

**Table:**

	*U*^11^	*U*^22^	*U*^33^	*U*^12^	*U*^13^	*U*^23^
C1	0.0615 (16)	0.0409 (12)	0.0393 (12)	−0.0039 (11)	0.0042 (11)	−0.0048 (10)
C2	0.077 (2)	0.0549 (15)	0.0662 (17)	−0.0057 (14)	0.0170 (14)	0.0055 (13)
C3	0.113 (3)	0.0589 (18)	0.088 (2)	−0.0037 (18)	0.029 (2)	0.0228 (16)
C4	0.122 (3)	0.0550 (17)	0.079 (2)	0.0146 (18)	0.009 (2)	0.0199 (15)
C5	0.085 (2)	0.0548 (16)	0.0655 (17)	0.0089 (15)	−0.0058 (15)	0.0032 (13)
C6	0.0648 (17)	0.0405 (13)	0.0448 (13)	−0.0022 (11)	−0.0040 (12)	−0.0036 (10)
C7	0.0530 (15)	0.0473 (13)	0.0444 (13)	−0.0065 (11)	−0.0049 (11)	0.0037 (10)
C8	0.0472 (15)	0.0441 (12)	0.0389 (12)	−0.0044 (11)	0.0013 (10)	−0.0014 (10)
C9	0.0485 (15)	0.0509 (13)	0.0418 (13)	−0.0049 (11)	0.0087 (11)	−0.0026 (10)
C10	0.0398 (13)	0.0415 (12)	0.0408 (12)	0.0001 (10)	0.0053 (9)	−0.0028 (9)
C11	0.0543 (16)	0.0471 (13)	0.0389 (12)	0.0006 (12)	0.0039 (11)	−0.0016 (10)
C12	0.0481 (15)	0.0512 (14)	0.0515 (14)	0.0070 (11)	0.0088 (11)	−0.0056 (11)
C13	0.0345 (12)	0.0399 (12)	0.0451 (12)	0.0002 (9)	0.0026 (9)	0.0006 (9)
C14	0.0343 (12)	0.0373 (11)	0.0405 (12)	−0.0001 (9)	0.0042 (9)	−0.0016 (9)
C15	0.0467 (16)	0.0659 (16)	0.0732 (18)	0.0148 (12)	−0.0017 (13)	0.0018 (13)
C16	0.0412 (14)	0.0379 (11)	0.0449 (13)	0.0015 (10)	0.0005 (10)	−0.0003 (10)
C17	0.0364 (13)	0.0438 (12)	0.0344 (11)	0.0027 (10)	−0.0006 (9)	0.0008 (9)
C18	0.0385 (14)	0.0508 (13)	0.0405 (12)	0.0051 (11)	−0.0012 (10)	−0.0010 (10)
C19	0.0507 (17)	0.0676 (17)	0.0611 (16)	0.0177 (13)	0.0089 (12)	−0.0044 (13)
C20	0.0408 (16)	0.095 (2)	0.0724 (18)	0.0072 (15)	0.0150 (13)	0.0071 (16)
C21	0.0399 (15)	0.0752 (18)	0.0701 (17)	−0.0080 (13)	0.0053 (13)	0.0116 (15)
C22	0.0421 (14)	0.0542 (14)	0.0483 (13)	−0.0060 (11)	−0.0020 (11)	0.0037 (11)
C23	0.0412 (14)	0.0433 (13)	0.0539 (14)	0.0019 (10)	−0.0023 (11)	0.0040 (11)
C24	0.0385 (14)	0.0616 (15)	0.0438 (13)	0.0058 (11)	−0.0035 (10)	0.0041 (11)
C25	0.0349 (13)	0.0492 (13)	0.0439 (12)	0.0052 (10)	−0.0032 (10)	−0.0029 (10)
C26	0.0485 (15)	0.0563 (15)	0.0562 (15)	0.0023 (11)	−0.0063 (11)	−0.0068 (12)
C27	0.0685 (19)	0.0746 (19)	0.0609 (18)	0.0085 (15)	−0.0121 (14)	−0.0219 (15)
C28	0.0685 (19)	0.100 (2)	0.0459 (15)	0.0131 (17)	−0.0011 (14)	−0.0111 (16)
C29	0.0514 (16)	0.090 (2)	0.0482 (15)	0.0054 (14)	0.0008 (12)	0.0055 (14)
C30	0.068 (2)	0.0710 (18)	0.0780 (19)	−0.0083 (14)	0.0082 (15)	0.0260 (15)
C31	0.075 (2)	0.090 (2)	0.082 (2)	−0.0139 (17)	0.0022 (16)	0.0151 (17)
C32	0.122 (4)	0.069 (2)	0.099 (3)	0.013 (2)	−0.021 (3)	−0.023 (2)
C33	0.141 (4)	0.100 (3)	0.104 (3)	−0.005 (3)	0.031 (4)	−0.025 (3)
S1	0.1168 (12)	0.0372 (5)	0.0811 (7)	0.0140 (6)	−0.0054 (7)	−0.0141 (4)
C32'	0.130 (6)	0.070 (5)	0.094 (5)	0.000 (5)	0.010 (5)	−0.030 (5)
C33'	0.131 (8)	0.086 (7)	0.111 (7)	0.001 (8)	0.019 (8)	−0.017 (7)
S1'	0.125 (5)	0.072 (4)	0.107 (5)	0.004 (4)	0.006 (5)	−0.020 (4)
N1	0.0474 (14)	0.0503 (12)	0.0558 (13)	−0.0038 (10)	−0.0075 (11)	0.0002 (9)
N2	0.0839 (17)	0.0565 (13)	0.0565 (13)	−0.0127 (12)	−0.0081 (11)	−0.0086 (11)
N3	0.0367 (11)	0.0452 (10)	0.0505 (11)	0.0051 (8)	0.0016 (8)	−0.0018 (8)
N4	0.0482 (13)	0.0377 (11)	0.0565 (12)	0.0047 (10)	0.0022 (9)	−0.0080 (9)
N5	0.0497 (12)	0.0499 (11)	0.0523 (12)	−0.0026 (9)	0.0026 (9)	0.0098 (9)
O1	0.0512 (11)	0.0866 (13)	0.0746 (12)	−0.0016 (9)	0.0207 (9)	0.0231 (10)
O2	0.0473 (10)	0.0415 (9)	0.0732 (11)	−0.0066 (7)	0.0078 (8)	0.0011 (8)
O3	0.0705 (12)	0.0417 (9)	0.0683 (11)	−0.0048 (8)	0.0002 (9)	−0.0053 (8)
O4	0.145 (2)	0.0573 (12)	0.0838 (15)	0.0281 (12)	−0.0163 (13)	−0.0165 (11)

### Geometric parameters (Å, º)

**Table:**

C1—C6	1.399 (3)	C20—H20	0.9300
C1—C2	1.401 (3)	C21—C22	1.378 (3)
C1—C8	1.442 (3)	C21—H21	0.9300
C2—C3	1.377 (4)	C22—H22	0.9300
C2—H2	0.9300	C23—O3	1.216 (3)
C3—C4	1.383 (5)	C23—N5	1.355 (3)
C3—H3	0.9300	C24—C29	1.373 (3)
C4—C5	1.368 (4)	C24—C25	1.391 (3)
C4—H4	0.9300	C24—N5	1.407 (3)
C5—C6	1.389 (3)	C25—C26	1.376 (3)
C5—H5	0.9300	C26—C27	1.397 (3)
C6—N1	1.380 (3)	C26—H26	0.9300
C7—N1	1.339 (3)	C27—C28	1.366 (4)
C7—C8	1.379 (3)	C27—H27	0.9300
C7—H7	0.9300	C28—C29	1.373 (4)
C8—C9	1.445 (3)	C28—H28	0.9300
C9—O1	1.219 (3)	C29—H29	0.9300
C9—C10	1.577 (3)	C30—N5	1.467 (3)
C10—C11	1.474 (3)	C30—C31	1.468 (4)
C10—C12	1.547 (3)	C30—H30A	0.9700
C10—C14	1.580 (3)	C30—H30B	0.9700
C11—N2	1.138 (3)	C31—H31A	0.9600
C12—N3	1.463 (3)	C31—H31B	0.9600
C12—H12A	0.9700	C31—H31C	0.9600
C12—H12B	0.9700	C32—S1	1.764 (4)
C13—N3	1.456 (3)	C32—H32A	0.9600
C13—C25	1.512 (3)	C32—H32B	0.9600
C13—C23	1.561 (3)	C32—H32C	0.9600
C13—C14	1.569 (3)	C33—S1	1.790 (5)
C14—C17	1.504 (3)	C33—H33A	0.9600
C14—C16	1.561 (3)	C33—H33B	0.9600
C15—N3	1.464 (3)	C33—H33C	0.9600
C15—H15A	0.9600	S1—O4	1.472 (2)
C15—H15B	0.9600	C32'—S1'	1.60 (3)
C15—H15C	0.9600	C32'—H32D	0.9600
C16—O2	1.218 (2)	C32'—H32E	0.9600
C16—N4	1.341 (3)	C32'—H32F	0.9600
C17—C22	1.382 (3)	C33'—S1'	1.70 (3)
C17—C18	1.391 (3)	C33'—H33D	0.9600
C18—C19	1.374 (3)	C33'—H33E	0.9600
C18—N4	1.394 (3)	C33'—H33F	0.9600
C19—C20	1.381 (4)	S1'—O4	1.871 (14)
C19—H19	0.9300	N1—H1A	0.84 (2)
C20—C21	1.371 (4)	N4—H4A	0.84 (3)
			
C6—C1—C2	118.1 (2)	C21—C20—H20	119.4
C6—C1—C8	107.0 (2)	C19—C20—H20	119.4
C2—C1—C8	134.9 (2)	C20—C21—C22	120.9 (2)
C3—C2—C1	118.1 (3)	C20—C21—H21	119.5
C3—C2—H2	120.9	C22—C21—H21	119.5
C1—C2—H2	120.9	C21—C22—C17	119.2 (2)
C2—C3—C4	122.1 (3)	C21—C22—H22	120.4
C2—C3—H3	118.9	C17—C22—H22	120.4
C4—C3—H3	118.9	O3—C23—N5	125.8 (2)
C5—C4—C3	121.5 (3)	O3—C23—C13	125.4 (2)
C5—C4—H4	119.2	N5—C23—C13	108.72 (19)
C3—C4—H4	119.2	C29—C24—C25	121.9 (2)
C4—C5—C6	116.4 (3)	C29—C24—N5	128.0 (2)
C4—C5—H5	121.8	C25—C24—N5	110.06 (19)
C6—C5—H5	121.8	C26—C25—C24	119.6 (2)
N1—C6—C5	128.8 (3)	C26—C25—C13	131.4 (2)
N1—C6—C1	107.4 (2)	C24—C25—C13	108.89 (19)
C5—C6—C1	123.7 (2)	C25—C26—C27	118.5 (2)
N1—C7—C8	110.6 (2)	C25—C26—H26	120.7
N1—C7—H7	124.7	C27—C26—H26	120.7
C8—C7—H7	124.7	C28—C27—C26	120.3 (3)
C7—C8—C1	105.5 (2)	C28—C27—H27	119.9
C7—C8—C9	129.2 (2)	C26—C27—H27	119.9
C1—C8—C9	125.2 (2)	C27—C28—C29	122.0 (3)
O1—C9—C8	121.9 (2)	C27—C28—H28	119.0
O1—C9—C10	118.1 (2)	C29—C28—H28	119.0
C8—C9—C10	119.94 (19)	C24—C29—C28	117.5 (3)
C11—C10—C12	109.13 (18)	C24—C29—H29	121.2
C11—C10—C9	106.63 (17)	C28—C29—H29	121.2
C12—C10—C9	112.08 (17)	N5—C30—C31	113.9 (2)
C11—C10—C14	112.47 (17)	N5—C30—H30A	108.8
C12—C10—C14	104.15 (16)	C31—C30—H30A	108.8
C9—C10—C14	112.44 (16)	N5—C30—H30B	108.8
N2—C11—C10	176.2 (2)	C31—C30—H30B	108.8
N3—C12—C10	106.32 (17)	H30A—C30—H30B	107.7
N3—C12—H12A	110.5	C30—C31—H31A	109.5
C10—C12—H12A	110.5	C30—C31—H31B	109.5
N3—C12—H12B	110.5	H31A—C31—H31B	109.5
C10—C12—H12B	110.5	C30—C31—H31C	109.5
H12A—C12—H12B	108.7	H31A—C31—H31C	109.5
N3—C13—C25	117.96 (18)	H31B—C31—H31C	109.5
N3—C13—C23	112.90 (17)	O4—S1—C32	108.70 (19)
C25—C13—C23	100.88 (17)	O4—S1—C33	105.2 (2)
N3—C13—C14	102.13 (16)	C32—S1—C33	97.3 (2)
C25—C13—C14	112.90 (16)	S1'—C32'—H32D	109.5
C23—C13—C14	110.34 (17)	S1'—C32'—H32E	109.5
C17—C14—C16	101.62 (16)	H32D—C32'—H32E	109.5
C17—C14—C13	112.62 (17)	S1'—C32'—H32F	109.5
C16—C14—C13	108.76 (16)	H32D—C32'—H32F	109.5
C17—C14—C10	121.29 (17)	H32E—C32'—H32F	109.5
C16—C14—C10	109.83 (16)	S1'—C33'—H33D	109.5
C13—C14—C10	102.46 (15)	S1'—C33'—H33E	109.5
N3—C15—H15A	109.5	H33D—C33'—H33E	109.5
N3—C15—H15B	109.5	S1'—C33'—H33F	109.5
H15A—C15—H15B	109.5	H33D—C33'—H33F	109.5
N3—C15—H15C	109.5	H33E—C33'—H33F	109.5
H15A—C15—H15C	109.5	C32'—S1'—C33'	94.8 (17)
H15B—C15—H15C	109.5	C32'—S1'—O4	76.2 (12)
O2—C16—N4	125.7 (2)	C33'—S1'—O4	114.4 (14)
O2—C16—C14	126.84 (19)	C7—N1—C6	109.5 (2)
N4—C16—C14	107.43 (19)	C7—N1—H1A	125.1 (17)
C22—C17—C18	118.7 (2)	C6—N1—H1A	125.3 (17)
C22—C17—C14	132.5 (2)	C13—N3—C12	107.13 (17)
C18—C17—C14	108.56 (18)	C13—N3—C15	114.81 (18)
C19—C18—C17	122.6 (2)	C12—N3—C15	112.82 (18)
C19—C18—N4	127.8 (2)	C16—N4—C18	112.51 (19)
C17—C18—N4	109.64 (19)	C16—N4—H4A	121.9 (17)
C18—C19—C20	117.4 (2)	C18—N4—H4A	124.8 (17)
C18—C19—H19	121.3	C23—N5—C24	110.86 (19)
C20—C19—H19	121.3	C23—N5—C30	123.2 (2)
C21—C20—C19	121.2 (2)	C24—N5—C30	125.8 (2)
			
C6—C1—C2—C3	−0.8 (4)	C22—C17—C18—N4	−179.31 (19)
C8—C1—C2—C3	178.4 (3)	C14—C17—C18—N4	−3.7 (2)
C1—C2—C3—C4	0.0 (4)	C17—C18—C19—C20	0.2 (4)
C2—C3—C4—C5	0.6 (5)	N4—C18—C19—C20	178.8 (2)
C3—C4—C5—C6	−0.2 (4)	C18—C19—C20—C21	0.1 (4)
C4—C5—C6—N1	−178.7 (2)	C19—C20—C21—C22	−0.3 (4)
C4—C5—C6—C1	−0.7 (4)	C20—C21—C22—C17	0.0 (4)
C2—C1—C6—N1	179.6 (2)	C18—C17—C22—C21	0.4 (3)
C8—C1—C6—N1	0.2 (2)	C14—C17—C22—C21	−174.0 (2)
C2—C1—C6—C5	1.2 (3)	N3—C13—C23—O3	45.5 (3)
C8—C1—C6—C5	−178.2 (2)	C25—C13—C23—O3	172.3 (2)
N1—C7—C8—C1	0.2 (2)	C14—C13—C23—O3	−68.1 (3)
N1—C7—C8—C9	179.1 (2)	N3—C13—C23—N5	−132.22 (19)
C6—C1—C8—C7	−0.3 (2)	C25—C13—C23—N5	−5.4 (2)
C2—C1—C8—C7	−179.5 (3)	C14—C13—C23—N5	114.21 (19)
C6—C1—C8—C9	−179.2 (2)	C29—C24—C25—C26	−4.0 (3)
C2—C1—C8—C9	1.5 (4)	N5—C24—C25—C26	175.3 (2)
C7—C8—C9—O1	−175.8 (2)	C29—C24—C25—C13	173.3 (2)
C1—C8—C9—O1	2.8 (3)	N5—C24—C25—C13	−7.4 (2)
C7—C8—C9—C10	5.0 (3)	N3—C13—C25—C26	−52.2 (3)
C1—C8—C9—C10	−176.37 (19)	C23—C13—C25—C26	−175.6 (2)
O1—C9—C10—C11	126.3 (2)	C14—C13—C25—C26	66.7 (3)
C8—C9—C10—C11	−54.5 (2)	N3—C13—C25—C24	130.97 (19)
O1—C9—C10—C12	6.9 (3)	C23—C13—C25—C24	7.6 (2)
C8—C9—C10—C12	−173.86 (18)	C14—C13—C25—C24	−110.2 (2)
O1—C9—C10—C14	−110.0 (2)	C24—C25—C26—C27	3.1 (3)
C8—C9—C10—C14	69.2 (2)	C13—C25—C26—C27	−173.4 (2)
C11—C10—C12—N3	126.58 (19)	C25—C26—C27—C28	−0.2 (4)
C9—C10—C12—N3	−115.54 (19)	C26—C27—C28—C29	−2.0 (4)
C14—C10—C12—N3	6.3 (2)	C25—C24—C29—C28	1.8 (4)
N3—C13—C14—C17	−168.92 (16)	N5—C24—C29—C28	−177.4 (2)
C25—C13—C14—C17	63.4 (2)	C27—C28—C29—C24	1.2 (4)
C23—C13—C14—C17	−48.6 (2)	C8—C7—N1—C6	−0.1 (3)
N3—C13—C14—C16	79.25 (19)	C5—C6—N1—C7	178.3 (2)
C25—C13—C14—C16	−48.4 (2)	C1—C6—N1—C7	−0.1 (2)
C23—C13—C14—C16	−160.48 (17)	C25—C13—N3—C12	167.45 (18)
N3—C13—C14—C10	−36.98 (18)	C23—C13—N3—C12	−75.4 (2)
C25—C13—C14—C10	−164.67 (17)	C14—C13—N3—C12	43.1 (2)
C23—C13—C14—C10	83.29 (19)	C25—C13—N3—C15	−66.4 (2)
C11—C10—C14—C17	27.0 (3)	C23—C13—N3—C15	50.7 (2)
C12—C10—C14—C17	145.03 (19)	C14—C13—N3—C15	169.20 (17)
C9—C10—C14—C17	−93.4 (2)	C10—C12—N3—C13	−31.4 (2)
C11—C10—C14—C16	145.00 (18)	C10—C12—N3—C15	−158.72 (18)
C12—C10—C14—C16	−96.96 (19)	O2—C16—N4—C18	−177.3 (2)
C9—C10—C14—C16	24.6 (2)	C14—C16—N4—C18	2.5 (2)
C11—C10—C14—C13	−99.54 (19)	C19—C18—N4—C16	−178.1 (2)
C12—C10—C14—C13	18.50 (19)	C17—C18—N4—C16	0.7 (3)
C9—C10—C14—C13	140.06 (17)	O3—C23—N5—C24	−176.3 (2)
C17—C14—C16—O2	175.5 (2)	C13—C23—N5—C24	1.4 (2)
C13—C14—C16—O2	−65.6 (3)	O3—C23—N5—C30	−0.3 (4)
C10—C14—C16—O2	45.8 (3)	C13—C23—N5—C30	177.3 (2)
C17—C14—C16—N4	−4.4 (2)	C29—C24—N5—C23	−176.9 (2)
C13—C14—C16—N4	114.59 (19)	C25—C24—N5—C23	3.8 (3)
C10—C14—C16—N4	−134.02 (18)	C29—C24—N5—C30	7.2 (4)
C16—C14—C17—C22	179.6 (2)	C25—C24—N5—C30	−172.1 (2)
C13—C14—C17—C22	63.4 (3)	C31—C30—N5—C23	102.0 (3)
C10—C14—C17—C22	−58.4 (3)	C31—C30—N5—C24	−82.7 (3)
C16—C14—C17—C18	4.8 (2)	C32—S1—O4—S1'	−131.2 (7)
C13—C14—C17—C18	−111.40 (19)	C33—S1—O4—S1'	−27.8 (6)
C10—C14—C17—C18	126.82 (19)	C32'—S1'—O4—S1	59.2 (13)
C22—C17—C18—C19	−0.5 (3)	C33'—S1'—O4—S1	148.3 (16)
C14—C17—C18—C19	175.1 (2)		

### Hydrogen-bond geometry (Å, º)

**Table:**

*D*—H···*A*	*D*—H	H···*A*	*D*···*A*	*D*—H···*A*
N4—H4*A*···O4	0.84 (3)	1.89 (3)	2.714 (3)	166 (2)
N1—H1*A*···O2^i^	0.84 (3)	2.10 (3)	2.887 (3)	155 (2)
C22—H22···S1^ii^	0.93	2.85	3.717 (3)	157
C32—H32*A*···O3^iii^	0.96	2.60	3.219 (4)	123

Symmetry codes: (i) *x*+1/2, *y*, −*z*+1/2; (ii) −*x*+3/2, *y*+1/2, *z*; (iii) −*x*+3/2, *y*−1/2, *z*.
